# Associations of self-reported physical activity, sedentary behavior, and sleep duration with mental and behavioral health among civil aviation flight students: a cross-sectional study

**DOI:** 10.3389/fpsyg.2026.1914711

**Published:** 2026-10-16

**Authors:** Yu Zhou, Yang Xu, Yanli He

**Affiliations:** 1 College of Aviation Physical Education, Civil Aviation Flight University of China, Chengdu, China; 2 School of Humanities Education and Application, Sichuan Technology and Business University, Chengdu, China

**Keywords:** civil aviation flight students, mental health, physical activity, sedentary behavior, sleep duration

## Abstract

**Objective:**

To examine the associations of self-reported physical activity at different intensities, total sedentary time, and sleep duration with mental and behavioral health indicators among civil aviation flight students.

**Methods:**

A cross-sectional questionnaire survey was conducted at a civil aviation university from January 5 to 30, 2026. A total of 776 students majoring in Flight Technology were included, of whom 774 (99.7%) were male. Because only two participants were female, the primary inferential analyses were restricted to male participants. Physical activity and sedentary behavior were assessed using modified self-report items. Physical activity duration represented participants’ self-reported activity time on a typical activity day rather than average daily or weekly physical activity time; sleep exposure was assessed using categorical self-reported sleep duration. Depression, anxiety, stress, and problematic Internet use were assessed using validated scales. Generalized estimating equation models were used to examine cross-sectional associations between the behavioral indicators and outcomes, with false discovery rate (FDR) correction.

**Results:**

In the fully adjusted male sample, after FDR correction, longer self-reported vigorous-intensity physical activity duration on a typical activity day was associated with lower anxiety scores; self-reported sleep duration <7 h was associated with higher depression, anxiety, stress, and problematic Internet use scores; and longer self-reported total sedentary time was associated with higher problematic Internet use scores. No statistically significant linear associations were observed between moderate- or light-intensity physical activity and any outcome after FDR correction. The observed associations were generally small: the association between vigorous-intensity physical activity and anxiety was approximately 0.10 outcome standard deviations, the associations between sleep duration <7 h and the corresponding outcomes were approximately 0.24–0.27 standard deviations, and the association between total sedentary time and problematic Internet use was approximately 0.04 standard deviations. The directions of the main associations were generally consistent across multiple sensitivity analyses, although the strength of statistical evidence for some associations varied by outcome definition, sample restriction, and data-processing approach.

**Conclusion:**

Among male civil aviation flight students, self-reported physical activity, total sedentary time, and sleep duration were cross-sectionally associated with selected mental and behavioral health indicators. Given the cross-sectional design, these findings cannot establish causality, an optimal physical activity dose, a sleep intervention threshold, or time-substitution effects between behaviors. The findings suggest that behavioral indicators and mental and behavioral health status may be considered jointly in this population, but the observed associations require confirmation in longitudinal studies using objective, device-based behavioral measures.

## 1 Introduction

Civil aviation flight students are an important source of future pilots for China’s civil aviation workforce (Hu et al., 2026). Compared with general university students, they must not only systematically study professional subjects such as aviation meteorology, principles of flight, and air traffic control, but also undergo practical flight training, licensing examinations, physical fitness requirements, and periodic aeromedical examinations while adapting to relatively strict daily management (Liao et al., 2025). These occupational-training demands may expose them to multiple psychological stressors (Yu et al., 2022). A nationwide study of 7,055 Chinese commercial pilots showed that 23.3 and 17.0% had depression and anxiety symptom scores above the corresponding screening thresholds, respectively, and that sleep duration <7 h was associated with higher risks of depression and anxiety (Chen et al., 2024). Therefore, attention to behavioral patterns and mental health among flight students is relevant to occupational training and health management (Zhao et al., 2024).

Physical activity, sedentary behavior, and sleep duration are all closely related to mental health. Previous studies generally indicate that higher levels of physical activity are associated with lower levels of depression, anxiety, and stress, whereas longer sedentary time and shorter sleep duration may be associated with poorer mental health (Tremblay et al., 2016; Martín-Rodríguez and González-Prieto, 2026; Huang et al., 2024; Jiang et al., 2020). Evidence from Chinese populations has also expanded substantially. A scoping review of 53 Chinese studies by Huang et al. (2024) showed rapid growth in this field, although the available evidence still relies mainly on cross-sectional designs and self-reported measures. Studies of guideline adherence have further found that, among Chinese adults, young adults, and university students, meeting a greater number of recommendations related to physical activity, sedentary behavior, and sleep is generally associated with lower depression and anxiety or better mental health (Duan et al., 2024; Lin et al., 2024; Yan et al., 2026). Meanwhile, several studies among Chinese university students have used compositional data analysis to examine relationships between the relative time allocation of physical activity, sedentary behavior, and sleep and depression, anxiety, and stress (Wang et al., 2023; Huang et al., 2025; Li et al., 2026; Dong et al., 2025). Thus, the contribution of the present study does not rest on an absence of relevant evidence among general Chinese university students.

A more important gap in the existing evidence concerns students receiving specialized aviation occupational training. The learning and training arrangements of civil aviation flight students may create group-specific behavioral patterns: theoretical coursework, self-study, simulator training, and preparation for assessments may increase sitting time, whereas physical education courses, physical fitness training, and occupational fitness requirements may influence physical activity levels; training schedules, daily routines, licensing examinations, and aeromedical examinations may also be related to both sleep and psychological status (Pan and Mendonca, 2025; Prokopczyk and Wochyński, 2022; Corbett and Newman, 2022; Boyd and Bliss, 2024). Therefore, findings from general university students or young adults may not be directly generalizable to civil aviation flight students. Evidence remains limited in this safety-critical occupational-training population regarding the simultaneous associations of physical activity, sedentary behavior, and sleep duration with multidimensional mental health outcomes. It should also be noted that the statistical framework used in the present study differs from those used in some previous studies. Guideline-adherence studies mainly examine whether predefined behavioral recommendations are met, whereas compositional data analysis reflects the relative allocation of behaviors within a finite 24-h time budget. In contrast, physical activity, sedentary behavior, and sleep duration in the present study were measured as separate self-reported indicators, and the analysis focused on their individual and mutually adjusted cross-sectional associations with the outcomes. Accordingly, the effect estimates cannot be interpreted as changes in 24-h behavioral composition or as effects of replacing time in one behavior with time in another. In addition to depression, anxiety, and stress, problematic Internet use is a behavioral health concern among university students and may be related to sedentary behavior, sleep, and physical activity patterns (Wang et al., 2025). Because problematic Internet use is conceptually distinct from depression, anxiety, and stress, it was examined as a supplementary behavioral health outcome.

Accordingly, we conducted a cross-sectional survey at a civil aviation university in January 2026 to examine the associations of self-reported vigorous-, moderate-, and light-intensity physical activity, total sedentary time, and sleep duration with depression, anxiety, stress, and problematic Internet use, with all behavioral indicators entered simultaneously in the fully adjusted model. The study aimed to characterize patterns of association between these behaviors and mental and behavioral health among male civil aviation flight students, a distinctive occupational-training population, and to provide a basis for future longitudinal studies and research using objective behavioral monitoring.

## 2 Participants and methods

### 2.1 Participants

This cross-sectional study was conducted as an online questionnaire survey at a civil aviation university from January 5 to 30, 2026. Participants were currently enrolled students majoring in Flight Technology and receiving civil aviation pilot training; students in other aviation-related majors were not included. The survey was administered by class. A total of 777 submitted questionnaires met the target-major eligibility criterion, and one questionnaire with an implausible reported age of 79 years was excluded, leaving 776 participants in the final sample. Participants were from the 2023, 2024, and 2025 admission cohorts and represented 56 class clusters. The original questionnaire did not separately record the specific stage of flight training; therefore, participants could not be further classified according to theoretical instruction, simulator training, or actual flight training.

The available study records did not fully preserve the original numbers of eligible or invited students and classes, the number of individuals who opened the questionnaire link, or the number who opened the link but did not submit a questionnaire; therefore, participation and completion rates could not be calculated reliably. Student ID was used as the primary identifier for potential duplicate submissions and was cross-checked against name, major and class, and submission time. One pair of records shared the same student ID, but the two questionnaires corresponded to different names and differed in individual information and responses; they were therefore treated as distinct submissions rather than duplicates. Completion time was used as a quality-control indicator, but no single *post hoc* duration threshold was used to automatically exclude questionnaires. Individual values that could not be reliably parsed were treated as missing rather than excluding an entire questionnaire because of a single abnormal value. The questionnaire included two instructional attention-check items. Performance on these items was not used as a direct exclusion criterion for the overall sample; instead, a sensitivity analysis restricted to participants who answered both items correctly was conducted. This study was approved by the Ethics Committee of China Civil Aviation Flight University (approval number: 20260051; approval date: January 2, 2026), and ethical approval was obtained before formal data collection. All participants provided informed consent before completing the questionnaire. For the four participants aged 17 years, informed consent was additionally obtained from their parents or legal guardians, and the participants themselves also provided informed consent. The study was conducted in accordance with the Declaration of Helsinki and its subsequent amendments.

### 2.2 Measures

#### 2.2.1 Physical activity, sedentary behavior, and sleep duration

Physical activity and sedentary behavior were assessed using self-administered modified items developed with reference to the general structure and activity-intensity concepts of the International Physical Activity Questionnaire–Short Form (IPAQ-SF) and tailored to daily activity contexts among university students (Craig et al., 2003). These items were neither standard IPAQ-SF items nor scored using the standard IPAQ-SF protocol; therefore, the resulting indicators should not be regarded as standard IPAQ-SF measures or considered directly equivalent to measures obtained using validated standardized questionnaires or accelerometers. The complete original items, activity examples, response formats, and variable-construction rules are provided in Supplementary Table S1. Physical activity intensity was classified according to the subjective intensity descriptions and activity examples in the original questionnaire rather than on the basis of measured energy expenditure or MET values. Vigorous-intensity physical activity mainly included activities that markedly increased breathing and heart rate and caused heavy sweating; moderate-intensity physical activity mainly included activities that moderately increased breathing and heart rate and caused mild sweating; and the light-intensity physical activity item included walking, standing activities, stretching, and other low-load activities. Because static standing does not necessarily meet an energy-expenditure-based definition of light-intensity physical activity, this indicator may involve some degree of activity-intensity construct misclassification and was therefore interpreted as a questionnaire-defined self-reported light-intensity physical activity indicator. For each physical activity intensity, the questionnaire also recorded duration on a typical activity day. Participants who did not engage in the corresponding activity were coded as 0 min, and activity time was converted to minutes using hours × 60 + minutes. Because the activity-frequency information available in the analytical database was insufficient to reliably calculate complete weekly physical activity volume, average daily physical activity duration, or MET-min/week, the three physical activity indicators represent only self-reported activity duration on a typical activity day. In the models, the regression coefficient per 30 min represents the difference in outcome score associated with a 30-min difference in duration on a typical activity day and does not represent the effect of increasing average daily or weekly physical activity time by 30 min.

Sedentary behavior was defined as behavior in a sitting or reclining posture while awake, excluding sleep (Tremblay et al., 2017). Sedentary time was reported directly by participants and converted to min/d; unmeasured remaining time was not back-calculated from physical activity or sleep duration. Thus, the physical activity, sedentary behavior, and sleep indicators in this study do not constitute an exhaustive and mutually exclusive 24-h closed time composition.

Sleep duration was assessed using an item developed with reference to the Pittsburgh Sleep Quality Index that asked about actual nightly sleep duration during the previous month. The original response options were ≥7 h, 6 to <7 h, 5 to <6 h, and <5 h (Buysse et al., 1989). All four categories were retained for descriptive analyses; for the primary regression analyses, they were collapsed into ≥7 h and <7 h, with ≥7 h as the reference category. Because the “≥7 h” category did not distinguish 7 to <8 h from ≥8 h, the available data did not permit reconstruction of a higher age-specific sleep threshold for the four 17-year-old participants. The binary classification was therefore retained in the primary analysis, and its influence was evaluated in a sensitivity analysis excluding participants aged <18 years. The sleep indicator represents self-reported sleep duration only and should not be interpreted as a measure of overall sleep health.

#### 2.2.2 Depression, anxiety, and stress

Depression, anxiety, and stress symptoms were assessed using the 21-item Simplified Chinese version of the Depression Anxiety Stress Scales (DASS-21) (Wang et al., 2016). The scale comprises three seven-item subscales. The original 1–4 response scores were converted to 0–3 before analysis; item scores within each subscale were summed and multiplied by 2, yielding a theoretical range of 0–42, with higher scores indicating more severe symptoms. According to the recommended cutoffs, scores of ≥10 for depression, ≥8 for anxiety, and ≥15 for stress were classified as being above the corresponding symptom screening thresholds. Because the DASS-21 is a symptom-screening instrument, these proportions were not interpreted as clinical prevalence or diagnostic rates. Cronbach’s α was used to assess the internal consistency of each subscale in the current sample.

#### 2.2.3 Problematic internet use

Problematic Internet use was assessed using the College Student Internet Dependence Measurement Tool revised by Bai and Fan (2005). The instrument comprises 19 items across four dimensions: compulsive Internet use and withdrawal, tolerance, interpersonal and health problems, and time-management problems. The original validation study, conducted among 388 university students from five universities in Beijing, reported internal consistency and validation evidence based on external indicators and groups with different levels of Internet use. Each item is rated on a 1–4 scale, yielding a total score of 19–76; higher scores indicate a greater tendency toward problematic Internet use. In the present study, problematic Internet use was treated as a supplementary behavioral health outcome. Cronbach’s α was 0.958 in the current sample; this coefficient was used only to assess internal consistency and was not treated as stand-alone evidence of construct validity.

#### 2.2.4 Covariates

Based on previous studies and the information available in the original database, age, body mass index, and admission year were included as covariates in the primary inferential models. Body mass index was calculated as body weight divided by height squared and expressed in kg/m^2^; admission year was determined from the year information contained in the major and class fields. Because only two participants were female, all 776 participants were included in the descriptive analyses, whereas the primary inferential analyses were restricted to the 774 male participants. Because the survey was organized by class, class code was used as the clustering variable to account for within-class correlation in the statistical models.

### 2.3 Statistical analysis

All data cleaning, variable construction, and statistical analyses were performed using R version 4.5.1. Participants who did not engage in the corresponding intensity of physical activity were coded as 0 min; text entries that could not be reliably converted, negative values, and single reported time values >1,440 min were treated as missing. In the primary analysis, values >180 min/typical activity day for each of the three physical activity indicators were truncated to 180 min. This truncation was used only to reduce the influence of extreme self-reported values and was not a standard IPAQ-SF scoring threshold. To evaluate its influence, the 95th and 99th percentiles, maximum values, and numbers and proportions of values >180 min before truncation were reported, and sensitivity analyses were performed using the original untruncated values and after excluding participants with any physical activity indicator >180 min. Height values <140 cm or >210 cm and weight values <35 kg or >200 kg were treated as missing in the relevant analyses. Complete-case analysis was used for all models, with no imputation of missing values. The numbers of valid observations, numbers and proportions of missing values, and known reasons for missingness for each exposure, outcome, and covariate are presented in Supplementary Table S2. Continuous variables were summarized using mean ± standard deviation and median (interquartile range), and categorical variables using number (percentage). The primary inferential analyses were restricted to male participants. Generalized estimating equations (GEE) were used to account for within-class correlation (Liang and Zeger, 1986), with class code as the clustering variable, an exchangeable working correlation structure, and robust sandwich standard errors. Depression, anxiety, stress, and problematic Internet use scores were treated as continuous outcomes and modeled using a Gaussian distribution with an identity link. The three physical activity variables were scaled per 30 min/typical activity day, sedentary behavior per 60 min/d, and sleep duration was modeled with ≥7 h as the reference category. Three models were fitted sequentially: Model 1 was an unadjusted model containing a single behavioral indicator; Model 2 adjusted for age, body mass index, and admission year; and Model 3 additionally included vigorous-, moderate-, and light-intensity physical activity, sedentary behavior, and sleep duration simultaneously to estimate their mutually adjusted cross-sectional associations with the outcomes. Results are reported as unstandardized regression coefficients (B), 95% CIs, and exact *p*-values. To facilitate interpretation of association magnitude, B coefficients from Model 3 were divided by the sample standard deviation of the corresponding outcome. Variance inflation factors were used to assess multicollinearity, and Pearson residuals were examined to assess model specification for continuous outcomes.

Given the potential for right-skewed distributions, floor effects, and substantial numbers of zero values in DASS-21 scores, the following sensitivity analyses were conducted: (1) the fully adjusted models were repeated after ln(*x* + 1) transformation of depression, anxiety, and stress scores; (2) binary indicators of scoring above the corresponding symptom screening thresholds were modeled using Poisson GEE to estimate prevalence ratios (PRs) and 95% CIs (Barros and Hirakata, 2003; Yelland et al., 2011); (3) untruncated physical activity durations were used; (4) participants with any physical activity indicator >180 min/typical activity day were excluded; (5) analyses were restricted to participants who answered both instructional attention-check items correctly; (6) an independent working correlation structure was used; and (7) participants aged <18 years were excluded. In exploratory analyses, quadratic terms for vigorous-, moderate-, and light-intensity physical activity and sedentary behavior were added separately to the fully adjusted models, resulting in 16 nonlinear tests. The 20 behavior–outcome associations in the primary analysis, the 15 associations in the DASS-21 log-transformed and symptom screening-threshold models, and the 16 exploratory quadratic-term tests were each subjected to FDR correction using the Benjamini–Hochberg method (Benjamini and Hochberg, 1995), with both raw *p* values and q values reported. A q value <0.05 was considered statistically significant after FDR correction. All effects were interpreted as cross-sectional statistical associations rather than as causal effects, behavioral time-substitution effects, or evidence of clinical importance.

## 3 Results

### 3.1 Participant characteristics

A total of 776 currently enrolled students majoring in Flight Technology and receiving civil aviation pilot training were included, of whom 774 (99.7%) were male and 2 (0.3%) were female. Participants were 17–21 years old, with a mean age of 18.93 ± 0.77 years; 4 participants (0.5%) were younger than 18 years. Participants were mainly from the 2024 and 2025 admission cohorts, accounting for 52.2 and 44.5%, respectively. A total of 56 class clusters were identified from the major and class information. Participant characteristics are shown in Table 1.

**Table 1:** Participant characteristics.

Variable	Overall (*n* = 776)
Sex	
Male	774(99.7)
Female	2(0.3)
Age, years	18.93 ± 0.77
Age range, years	17–21
Height, cm	177.54 ± 4.79
Weight, kg	71.20 ± 13.77
Body mass index, kg/m^2^	22.58 ± 4.21
Admission year	
2023 cohort	17(2.2)
2024 cohort	405(52.2)
2025 cohort	345(44.5)
Unidentified	9(1.2)
Class clusters, *n*	56
Age <18 years, *n* (%)	4(0.5)

Continuous variables are presented as mean ± standard deviation, and categorical variables as number (percentage). Height, weight, and body mass index were calculated from 774, 775, and 773 valid observations, respectively. Percentages may not sum to 100.0% because of rounding. All participants were currently enrolled students majoring in Flight Technology and receiving civil aviation pilot training.

### 3.2 Descriptive characteristics of physical activity, sedentary behavior, sleep duration, and mental and Behavioral health indicators

The median durations of vigorous-, moderate-, and light-intensity physical activity were 90, 80, and 90 min/typical activity day, respectively, and the median directly reported sedentary time was 320 min/d. Before applying the 180 min/typical activity day truncation rule used in the primary analysis, 27 (3.5%), 30 (3.9%), and 127 (16.4%) participants reported >180 min of vigorous-, moderate-, and light-intensity physical activity, respectively. The corresponding 95th percentiles were 172.7, 180.0, and 301.4 min; the 99th percentiles were 240.0, 247.5, and 482.8 min; and the maximum values were 360, 360, and 960 min, respectively. Overall, 150 participants (19.3%) had at least one physical activity indicator >180 min/typical activity day. The upper-tail distributions of the physical activity indicators before truncation are shown in Supplementary Table S3.

A total of 411 participants (53.0%) reported sleep duration ≥7 h and 365 (47.0%) reported sleep duration <7 h. The median depression, anxiety, and stress scores were 0, 2, and 0, respectively; 541 (69.7%), 383 (49.4%), and 429 (55.3%) participants, respectively, had scores of 0, indicating varying degrees of floor effects and right-skewed distributions. The distributions of the outcome scores are shown in Supplementary Figures 1–4. The numbers of participants scoring above the corresponding symptom screening thresholds were 51 (6.6%), 87 (11.2%), and 13 (1.7%), respectively. The mean problematic Internet use score was 30.05 ± 9.14. Cronbach’s α coefficients for the depression, anxiety, and stress subscales and the problematic Internet use scale were 0.845, 0.764, 0.835, and 0.958, respectively. Detailed results are shown in Table 2.

**Table 2:** Descriptive characteristics of physical activity, sedentary behavior, sleep duration, and mental and behavioral health indicators.

Category	Indicator	Mean ± SD	Median (P25, P75)	Count or zero value, *n* (%)	Above screening threshold, *n* (%)	Cronbach’s *α*
Activity behaviors	Vigorous-intensity physical activity, min/typical activity day	84.45 ± 47.10	90(60, 120)	0 min: 61(7.9)	–	–
	Moderate-intensity physical activity, min/typical activity day	82.05 ± 46.47	80(60, 120)	0 min: 44(5.7)	–	–
	Light-intensity physical activity, min/typical activity day	101.86 ± 54.34	90(60, 150)	0 min: 17(2.2)	–	–
	Sedentary behavior, min/d	345.51 ± 208.28	320(180, 480)	0 min: 2(0.3)	–	–
Sleep duration	≥7 h	–	–	411(53.0)	–	–
	6–<7 h	–	–	342(44.1)	–	–
	5–<6 h	–	–	21(2.7)	–	–
	<5 h	–	–	2(0.3)	–	–
Mental health	Depression	1.63 ± 3.48	0(0, 2)	Score of 0: 541 (69.7)	51(6.6)	0.845
	Anxiety	2.42 ± 3.64	2(0, 4)	Score of 0: 383 (49.4)	87(11.2)	0.764
	Stress	2.72 ± 4.32	0(0, 4)	Score of 0: 429 (55.3)	13(1.7)	0.835
	Problematic Internet use	30.05 ± 9.14	29(21, 38)	–	–	0.958

Continuous variables are presented as both mean ± standard deviation and median (25th percentile, 75th percentile), and categorical variables as number (percentage). Vigorous-, moderate-, and light-intensity physical activity durations represent participants’ self-reported activity time on a typical activity day rather than average daily or weekly physical activity duration; descriptive statistics for the three physical activity indicators in the table are based on the variables truncated at 180 min/typical activity day in the primary analysis; upper-tail distributions before truncation are shown in Supplementary Table S3. The screening thresholds for depression, anxiety, and stress were ≥10, ≥8, and ≥15, respectively. Vigorous-, moderate-, and light-intensity physical activity were calculated from 775, 776, and 773 valid observations, respectively; sedentary behavior was calculated from 772 valid observations, and all other indicators from 776 observations.

### 3.3 Associations of physical activity, sedentary behavior, and sleep duration with mental and behavioral health outcomes

The fully adjusted model for male participants included 756 participants and 48 class clusters. After accounting for within-class correlation and adjusting for age, body mass index, admission year, and the other measured behavioral indicators, each 30-min difference in self-reported vigorous-intensity physical activity duration on a typical activity day was associated with a 0.356-point lower anxiety score (*B* = −0.356, 95% CI: −0.562 to −0.150, *p* < 0.001, *q* = 0.005). Compared with participants reporting sleep duration ≥7 h, those reporting <7 h had depression, anxiety, stress, and problematic Internet use scores that were higher by 0.912, 0.981, 1.095, and 2.195 points, respectively; all four associations remained statistically significant after FDR correction (*q* ≤ 0.008). Each 60-min difference in sedentary time was associated with a 0.324-point higher problematic Internet use score (95% CI: 0.108 to 0.540, *p* = 0.003, *q* = 0.011). None of the remaining associations reached statistical significance after FDR correction (Table 3).

**Table 3:** Generalized estimating equation analysis of the associations of physical activity, sedentary behavior, and sleep duration with mental and behavioral health outcomes.

Outcome	Behavioral indicator	Model 1: B (95% CI), *p*-value	Model 2: B (95% CI), *P*-value	Model 3: B (95% CI), *P*-value	*q*-value
Depression	Vigorous-intensity physical activity, per 30 min/typical activity day	−0.174(−0.340−−0.008), 0.040	−0.164(−0.332–0.004), 0.056	−0.225(−0.433−−0.016), 0.035	0.087
	Moderate-intensity physical activity, per 30 min/typical activity day	0.032(−0.156–0.220), 0.738	0.032(−0.159–0.223), 0.742	0.129(−0.092–0.349), 0.252	0.337
	Light-intensity physical activity, per 30 min/typical activity day	0.002(−0.131–0.135), 0.978	0.012(−0.123–0.146), 0.866	0.007(−0.129–0.143), 0.919	0.919
	Sedentary behavior, per 60 min/d	0.019(−0.057–0.095), 0.626	0.003(−0.073–0.079), 0.939	−0.006(−0.083–0.071), 0.879	0.919
	Sleep duration <7 h (reference: ≥7 h)	0.871(0.335–1.407), 0.001	0.885(0.359–1.412), <0.001	0.912(0.343–1.481), 0.002	0.008
Anxiety	Vigorous-intensity physical activity, per 30 min/typical activity day	−0.261(−0.432−−0.090), 0.003	−0.268(−0.443−−0.092), 0.003	−0.356(−0.562−−0.150), <0.001	0.005
	Moderate-intensity physical activity, per 30 min/typical activity day	0.018(−0.166–0.202), 0.851	0.017(−0.174–0.208), 0.861	0.181(−0.048–0.410), 0.122	0.222
	Light-intensity physical activity, per 30 min/typical activity day	−0.028(−0.154–0.098), 0.667	−0.021(−0.151–0.108), 0.749	−0.019(−0.153–0.116), 0.786	0.873
	Sedentary behavior, per 60 min/d	0.063(−0.019–0.145), 0.134	0.059(−0.023–0.142), 0.158	0.054(−0.027–0.134), 0.191	0.294
	Sleep duration <7 h (reference: ≥7 h)	0.984(0.455–1.514), <0.001	0.987(0.456–1.517), <0.001	0.981(0.439–1.523), <0.001	0.004
Stress	Vigorous-intensity physical activity, per 30 min/typical activity day	−0.233(−0.440−−0.026), 0.028	−0.213(−0.429–0.002), 0.052	−0.284(−0.540−−0.029), 0.029	0.084
	Moderate-intensity physical activity, per 30 min/typical activity day	−0.003(−0.241–0.236), 0.983	0.013(−0.232–0.258), 0.916	0.150(−0.143–0.443), 0.317	0.396
	Light-intensity physical activity, per 30 min/typical activity day	−0.039(−0.169–0.091), 0.556	−0.023(−0.153–0.107), 0.729	−0.032(−0.166–0.102), 0.643	0.756
	Sedentary behavior, per 60 min/d	0.110(0.009–0.212), 0.033	0.093(−0.008–0.195), 0.072	0.085(−0.015–0.185), 0.094	0.209
	Sleep duration <7 h (reference: ≥7 h)	1.115(0.553–1.677), <0.001	1.141(0.571–1.711), <0.001	1.095(0.516–1.675), <0.001	0.004
Problematic Internet use	Vigorous-intensity physical activity, per 30 min/typical activity day	−0.588(−0.897−−0.278), <0.001	−0.509(−0.836−−0.182), 0.002	−0.348(−0.783–0.086), 0.116	0.222
	Moderate-intensity physical activity, per 30 min/typical activity day	−0.587(−0.956−−0.218), 0.002	−0.517(−0.876−−0.159), 0.005	−0.272(−0.734–0.189), 0.248	0.337
	Light-intensity physical activity, per 30 min/typical activity day	−0.383(−0.652−−0.114), 0.005	−0.345(−0.607−−0.083), 0.010	−0.210(−0.515–0.095), 0.177	0.294
	Sedentary behavior, per 60 min/d	0.365(0.137–0.593), 0.002	0.339(0.119–0.558), 0.003	0.324(0.108–0.540), 0.003	0.011
	Sleep duration <7 h (reference: ≥7 h)	2.185(0.844–3.527), 0.001	2.435(1.046–3.824), <0.001	2.195(0.804–3.587), 0.002	0.008

The primary inferential analyses were restricted to male participants. Model 1 was an unadjusted model containing a single behavioral indicator; Model 2 adjusted for age, body mass index, and admission year; and Model 3 additionally included vigorous-, moderate-, and light-intensity physical activity, sedentary behavior, and sleep duration simultaneously. All three physical activity indicators represent participants’ self-reported activity duration on a typical activity day. In the primary analysis, values >180 min/typical activity day were truncated to 180 min, and regression coefficients are interpreted per 30 min/typical activity day. Sedentary behavior is interpreted per 60 min/d, and sleep duration uses ≥7 h as the reference category. Model 3 included 756 male participants and 48 class clusters. B denotes the unstandardized regression coefficient and CI denotes confidence interval; q values are the Benjamini–Hochberg-corrected results for the 20 primary associations in Model 3. All estimates represent cross-sectional statistical associations and are not interpreted as causal effects or behavioral time-substitution effects.

In terms of association magnitude, a 30-min difference in vigorous-intensity physical activity duration on a typical activity day corresponded to an approximately 0.10-standard-deviation lower anxiety score; sleep duration <7 h corresponded to approximately 0.24–0.27-standard-deviation higher depression, anxiety, stress, and problematic Internet use scores; and a 60-min difference in sedentary time corresponded to an approximately 0.04-standard-deviation higher problematic Internet use score. These standardized differences are provided only to aid interpretation of association magnitude, do not indicate clinical importance, and should not be interpreted as causal effects of behavioral change.

### 3.4 Model diagnostics and sensitivity analyses

Variance inflation factors for the five behavioral indicators in the fully adjusted model ranged from 1.03 to 1.42, indicating no obvious multicollinearity, and the absolute values of the estimated within-class working correlation coefficients were <0.01 for all outcome models. In the male primary analysis, Pearson residuals for the depression, anxiety, and stress models showed varying degrees of right skew, whereas the residual distribution for the problematic Internet use model was relatively symmetric; the corresponding diagnostic plots are shown in Supplementary Figures 5–8. Given the floor effects and skewed distributions of the DASS-21 scores, alternative outcome specifications were used to assess sensitivity to model specification. After ln(*x* + 1) transformation of the DASS-21 scores, the associations of vigorous-intensity physical activity with anxiety and of sleep duration <7 h with depression, anxiety, and stress remained statistically significant after FDR correction; all 15 results are presented in Supplementary Table S4. When symptom screening thresholds were modeled as binary outcomes, 51, 86, and 13 participants in the fully adjusted male analysis were above the depression, anxiety, and stress screening thresholds, respectively. Only the association of vigorous-intensity physical activity with being above the anxiety screening threshold (PR = 0.810, 95% CI: 0.719–0.912, *q* = 0.008) and the association of sleep duration <7 h with being above the depression screening threshold (PR = 2.286, 95% CI: 1.264–4.135, *q* = 0.047) remained statistically significant after FDR correction. All 15 results are presented in Supplementary Table S5. Because some outcomes had few above-threshold cases, particularly stress (*n* = 13), these models were used only as sensitivity analyses and were not interpreted as confirmatory evidence of robustness. When the analysis was repeated using the original untruncated physical activity durations, all six FDR-significant associations from the primary analysis retained the same direction and remained at *q* < 0.05. After excluding 150 male participants with any physical activity indicator >180 min/typical activity day, the fully adjusted model included 608 participants and 48 class clusters; the same six primary associations retained the same direction and remained at *q* < 0.05. In this sensitivity analysis, the positive association between sedentary time and stress score also reached statistical significance after FDR correction (*B* = 0.148, 95% CI: 0.027–0.268, *p* = 0.016, *q* = 0.048), whereas it did not meet the corresponding criterion in the primary analysis, suggesting that the statistical significance of some secondary associations may be sensitive to the handling of extreme values. Complete results are presented in Supplementary Tables S6, S7.

The exploratory nonlinear analysis comprised 16 quadratic-term tests. The uncorrected *p*-values for the quadratic term of light-intensity physical activity in relation to depression, anxiety, and stress were 0.011, <0.001, and 0.014, respectively. After FDR correction, only the association with anxiety remained statistically significant (*q* = 0.001), whereas those with depression and stress did not (both *q* = 0.073). No other quadratic terms were statistically significant after FDR correction; complete results are presented in Supplementary Table S8. Given the exploratory nature of this analysis and the potential construct misclassification of light-intensity physical activity, these findings were not used as the basis for primary inference. After restricting the analysis to male participants who correctly answered both instructional attention-check items, the fully adjusted model included 674 participants and 46 class clusters. When the models were refitted using an independent working correlation structure, the six main associations retained the same direction; complete results are presented in Supplementary Tables S9, S10, respectively. After additionally excluding 4 male participants aged 17 years, the fully adjusted model included 752 participants and 48 class clusters. All six FDR-significant associations from the primary analysis retained the same direction and remained at *q* < 0.05, and the four sleep-duration associations did not change materially; the complete set of 20 results is presented in Supplementary Table S11.

## 4 Discussion

### 4.1 Association between vigorous-intensity physical activity and anxiety

In the male primary inferential sample, longer self-reported vigorous-intensity physical activity duration on a typical activity day was associated with lower anxiety scores, and the association remained statistically significant after FDR correction. The magnitude of this association was approximately 0.10 outcome standard deviations, indicating a small magnitude. This finding is directionally consistent with recent studies among Chinese university students. Using a prospective compositional data analysis, Wang et al. (2023) found that greater relative time in moderate-to-vigorous physical activity was associated with lower levels of depression, anxiety, and stress; Li et al. (2026) found that greater relative time in moderate-to-vigorous physical activity was associated with lower depression and stress; and Yan et al. (2026) reported that meeting a greater number of physical activity, sedentary behavior, and sleep recommendations was associated with lower depression, anxiety, and stress symptoms. However, those studies used compositional data analysis or guideline-adherence frameworks, whereas the present study used modified self-report items and estimated mutually adjusted cross-sectional associations among separately measured behavioral indicators. Their effect estimates are therefore not directly comparable with those from the present study and cannot be interpreted as behavioral time-substitution effects. Vigorous-intensity physical activity was also negatively associated with depression and stress in the present study, but these associations did not remain statistically significant after FDR correction. Accordingly, the current findings do not support a consistent association of vigorous-intensity physical activity across different dimensions of mental health. The cross-sectional design also cannot exclude reverse causation, whereby students with lower anxiety may be more likely to engage in vigorous-intensity physical activity, or residual confounding by unmeasured factors such as physical fitness and training habits.

### 4.2 Associations of self-reported sleep duration with mental and behavioral health outcomes

Sleep duration showed relatively consistent associations across multiple outcomes. Compared with participants reporting sleep duration ≥7 h, those reporting <7 h had higher depression, anxiety, stress, and problematic Internet use scores, with association magnitudes of approximately 0.24–0.27 outcome standard deviations. After excluding the four 17-year-old participants, the directions of these associations and the FDR-corrected results did not materially change. Recent studies among Chinese university students and young adults generally support an association between sleep and mental health. Wang et al. (2023) found that greater relative sleep time was associated with lower depression, anxiety, and stress; Li et al. (2026) reported that greater relative sleep time was associated with lower anxiety and stress; and guideline-adherence studies by Lin et al. (2024) and Yan et al. (2026) showed that meeting more recommendations for sleep and other activity behaviors was associated with better mental health. Huang et al. (2025), using compositional data analysis with accelerometer-measured activity combined with self-reported sleep duration, also observed an association between 24-h behavioral time composition and depressive symptoms. However, these studies differ from the present study in design, measurement, and statistical framework. In particular, compositional data analysis reflects relative time allocation among behaviors within a finite 24-h time budget, whereas the present study estimates mutually adjusted cross-sectional associations among separately measured behavioral indicators. The regression coefficient for sleep duration <7 h therefore cannot be interpreted as the effect of extending sleep while reducing other behaviors by an equivalent amount.

Across sensitivity analyses, the directions of the main sleep associations were generally preserved. After log transformation of DASS-21 scores, the associations of sleep duration <7 h with depression, anxiety, and stress remained statistically significant after FDR correction; in the symptom screening-threshold models, only the association with being above the depression screening threshold remained statistically significant after FDR correction. After excluding participants with any physical activity indicator >180 min/typical activity day, the association between sleep duration <7 h and depression score remained statistically significant (*B* = 0.838, 95% CI: 0.152–1.524, *q* = 0.048), indicating a relatively consistent direction of association, although the strength of statistical evidence varied by outcome definition and model specification. It is important to emphasize that the sleep exposure consisted only of categorical self-reported sleep duration. Although the original questionnaire contained additional sleep-related items, these dimensions were not incorporated into the current sleep-exposure construct or statistical models, and sleep regularity was not specifically measured. The findings therefore cannot be interpreted as associations with overall sleep health. Likewise, the association between sleep duration <7 h and problematic Internet use does not establish temporal ordering or determine whether unmeasured factors such as nighttime screen use contributed to the observed association.

### 4.3 Associations of sedentary behavior, problematic internet use, and other activity behaviors

Longer self-reported total sedentary time was associated with higher problematic Internet use scores, but the magnitude of the association was only approximately 0.04 outcome standard deviations and was therefore small. The present study measured total sedentary time and did not distinguish specific sedentary contexts such as study, transportation, or leisure, nor did it separately measure screen-based sedentary behavior. Total sedentary time therefore cannot be regarded as a proxy for screen exposure or screen-based sedentary behavior. The association between total sedentary time and problematic Internet use may partly reflect overlap between the two behaviors in digital-media-use contexts; however, because the study did not collect data on screen time, device use, or specific sedentary contexts, the extent of such overlap cannot be determined, and the findings cannot be used to infer that reducing total sedentary time would improve problematic Internet use.

No statistically significant linear association between light-intensity physical activity and stress was observed after FDR correction, which differs from the findings of Dong et al., who used accelerometry and compositional data analysis (Dong et al., 2025). This discrepancy may reflect differences in measurement methods and analytical frameworks. In the present study, light-intensity physical activity was derived from modified self-report items that included walking, standing, stretching, and other activities. Because static standing does not necessarily meet an energy-expenditure-based definition of light-intensity physical activity, this indicator may involve some degree of construct misclassification. No statistically significant linear associations were observed between moderate- or light-intensity physical activity and most outcomes after FDR correction. In the exploratory analysis, only the quadratic term for light-intensity physical activity in relation to anxiety remained statistically significant after FDR correction. Given the exploratory nature of the analysis and the potential construct misclassification of light-intensity physical activity, this finding should be regarded as hypothesis-generating and should not be used to determine an optimal activity level or dose threshold.

### 4.4 Outcome distributions, nonlinearity, and consistency of results

DASS-21 depression, anxiety, and stress scores showed pronounced floor effects, substantial numbers of zero values, and right-skewed distributions, and Pearson residuals also indicated residual skewness in some models. Accordingly, outcome transformations, symptom screening-threshold models, and multiple sensitivity analyses were used to assess the sensitivity of the findings to model specification. Overall, the directions of the main associations were largely consistent across analyses, although statistical significance varied. In the symptom screening-threshold models, 51, 86, and 13 participants were above the depression, anxiety, and stress screening thresholds, respectively. The small number of participants above the stress threshold limited the precision of the corresponding PR estimates; these analyses therefore should not be interpreted as confirming the robustness of the primary findings. When either untruncated data were used or participants with any physical activity indicator >180 min were excluded, the six FDR-significant associations from the primary analysis retained the same direction and remained at *q* < 0.05. However, in the extreme-value exclusion analysis, the association between sedentary time and stress also reached statistical significance after FDR correction (*B* = 0.148, 95% CI: 0.027–0.268, *q* = 0.048), whereas it did not meet the corresponding criterion in the primary analysis. This finding indicates that the statistical significance of some secondary associations may remain sensitive to data-processing choices. In the exploratory quadratic-term analysis, only the quadratic association between light-intensity physical activity and anxiety remained statistically significant after FDR correction (*q* = 0.001), whereas those with depression and stress did not (both *q* = 0.073). A scoping review by Huang et al. (2024) of 53 Chinese studies of 24-h movement behaviors indicated that the existing literature remains dominated by cross-sectional designs and self-reported measurements. Given the cross-sectional design, self-reported exposures, distributional characteristics of the DASS-21 outcomes, and variation in some results across model specifications, the present study is best interpreted as describing patterns of association among male civil aviation flight students and should not be used to infer causality, time-substitution effects, dose thresholds, or intervention targets.

### 4.5 Study significance and practical implications

The present study identified differing patterns of association between self-reported behavioral indicators and mental and behavioral health outcomes among male civil aviation flight students, but the associations were generally small in magnitude; in particular, the association between total sedentary time and problematic Internet use was only approximately 0.04 outcome standard deviations. Statistical significance should therefore not be equated directly with substantial clinical or practical importance. The findings suggest that physical activity participation, sleep duration, total sedentary time, and mental health status may be considered jointly in health monitoring and risk-identification efforts involving male civil aviation flight students. However, this study cannot determine an optimal physical activity dose, establish a causal benefit of vigorous-intensity physical activity, identify a sleep intervention threshold, or support specific behavioral prescriptions. Future studies should use longitudinal designs to clarify the temporal ordering between behaviors and mental health and incorporate objective measures such as accelerometers and wearable devices to more accurately assess physical activity, sedentary behavior, and sleep characteristics, while also considering specific sedentary contexts, stage of flight training, and other potential confounders.

### 4.6 Limitations

This study has several limitations. First, the cross-sectional design cannot determine the temporal ordering or causal relationships between physical activity, sedentary behavior, sleep duration, and mental and behavioral health outcomes, and reverse causation cannot be excluded. Second, physical activity was assessed using self-administered modified items developed with reference to the general structure of the IPAQ-SF rather than standard IPAQ-SF items, and the standard IPAQ-SF scoring protocol was not used. The activity-frequency information available in the analytical database was insufficient to reliably calculate complete weekly physical activity volume, average daily activity duration, or MET-min/week; therefore, each 30-min unit in the models represents only a difference in self-reported duration on a typical activity day. The light-intensity physical activity item also included walking, standing, and stretching and may therefore involve some degree of construct misclassification. All behavioral indicators may be affected by self-report bias. The primary sleep analysis used the categories ≥7 h and <7 h. The 4 participants aged 17 years may have been subject to age-specific classification uncertainty, although excluding them did not materially change the main sleep associations. The 7-h cutoff reflects only the analytical grouping used in this study and should not be interpreted as a validated intervention threshold. Third, truncation at 180 min/typical activity day was an analytical choice intended only to reduce the influence of extreme self-reported values and was not a standard IPAQ-SF scoring threshold or physiological cutoff. Although the main associations retained the same direction under two alternative approaches to handling extreme values, the statistical significance of some secondary associations changed, indicating that the results may remain sensitive to data-processing choices. Fourth, the physical activity, sedentary behavior, and sleep indicators did not constitute an exhaustive and mutually exclusive 24-h closed time composition; therefore, the findings cannot be interpreted in terms of compositional data analysis or time substitution. The sleep exposure also represented only self-reported sleep duration rather than overall sleep health. Fifth, although the primary models adjusted for age, body mass index, admission year, and other measured behavioral indicators, social support, academic workload, stage of flight training, screen time, timing of Internet use, previous mental health status, and other sleep characteristics were not included; residual confounding therefore remains possible. Sixth, the primary inferential analyses were restricted to male participants, and the two female participants were included only in the descriptive analyses; therefore, the findings cannot be generalized to female civil aviation flight students. The sample was drawn from a single civil aviation university, which also limits generalizability to students at other institutions and in other pilot-training systems. In addition, the DASS-21 outcomes showed marked skewness and floor effects, and the number of participants above some screening thresholds was small. Although multiple sensitivity analyses generally supported the directions of the main associations, they cannot eliminate all uncertainty related to model specification and measurement.

## 5 Conclusion

Among male civil aviation flight students, self-reported physical activity, total sedentary time, and sleep duration were cross-sectionally associated with selected mental and behavioral health indicators. Longer self-reported vigorous-intensity physical activity duration on a typical activity day was associated with lower anxiety scores; sleep duration <7 h was associated with higher depression, anxiety, stress, and problematic Internet use scores; and longer total sedentary time was associated with higher problematic Internet use scores. No statistically significant linear associations were observed between moderate- or light-intensity physical activity and any outcome after FDR correction. The directions of the main associations were generally consistent across multiple sensitivity analyses, although the strength of statistical evidence for some findings varied by outcome definition and model specification. Given the cross-sectional design and self-reported behavioral indicators, these findings cannot establish causality, an optimal physical activity dose, a sleep intervention threshold, or behavioral time-substitution effects. The findings support joint consideration of physical activity, total sedentary time, sleep duration, and mental health status among male civil aviation flight students, but require further confirmation through longitudinal research and objective behavioral monitoring. The findings should not be generalized to female civil aviation flight students.

## Data Availability

The raw data supporting the conclusions of this article will be made available by the authors, without undue reservation.
