# Supplementary material for: Associations of self-reported physical activity, sedentary behavior, and sleep duration with mental and behavioral health among civil aviation flight students: a cross-sectional study
Source: Front Psychol. 2026 Oct 16;17:1914711. doi: 10.3389/fpsyg.2026.1914711 (PMC13719623; doi:10.3389/fpsyg.2026.1914711)
Supplement: Supplementary file 1 [file Supplementary_file_1.DOCX]

**Supplementary Table S1. Original Questionnaire Items, Response Formats, and Construction of Analysis Variables for Physical Activity, Sedentary Behavior, and Sleep Duration**

| **Analysis indicator** | **Original questionnaire item** | **Original response/recording format** | **Variable construction and interpretation in this study** |
| --- | --- | --- | --- |
| Vigorous-intensity physical activity participation | 1. During the past 7 days, on how many days did you engage in vigorous (high-intensity) physical activity? (Definition: activities that make you breathe hard, markedly increase your heart rate, cause heavy sweating, and make it difficult to speak continuously.) Examples include: exercise: running (5 km/10 km/jogging), swimming, rope skipping, HIIT, vigorous aerobics; competitive ball sports: basketball, football, singles tennis/badminton; other activities: fast cycling (when in a hurry or uphill), carrying heavy objects upstairs (e.g., water buckets/heavy boxes of books). (Note: Do not include ordinary walking or slow jogging.) | The original database retained only categorical coding for this item and did not retain the complete number of days required to calculate a 0–7 d activity frequency. | Used to assist in determining whether the corresponding intensity of physical activity was performed; not used to calculate weekly physical activity volume, average daily physical activity time, or MET-min/week. |
| Vigorous-intensity physical activity duration | 2. On one of these days, how much time do you usually spend in vigorous (high-intensity) physical activity? (If the duration differs from day to day, please estimate the average number of hours and minutes, or use records from a fitness tracker if available.) | “____ hours per day”; “____ minutes (less than 60 minutes)” | Hours and minutes were converted to minutes. For participants engaging in the corresponding activity, duration was calculated as hours × 60 + minutes; participants who did not engage in the activity were coded as 0 min. The final variable was interpreted as self-reported vigorous-intensity physical activity duration on a typical activity day, min/typical activity day. |
| Moderate-intensity physical activity participation | 3. During the past 7 days, on how many days did you engage in moderate-intensity physical activity? (Definition: activities that make your breathing and heart rate increase slightly and cause mild sweating; you can talk but cannot sing.) Please include: exercise: jogging, yoga, Pilates, doubles tennis/badminton (recreational), skateboarding; transportation: cycling (usual speed, not uphill), brisk walking while carrying a load (e.g., carrying a backpack while hurrying to class); daily living: strenuous dormitory cleaning (mopping floors, cleaning windows), carrying light objects. (Note: Do not include ordinary walking.) | The original database retained only categorical coding for this item and did not retain the complete number of days required to calculate a 0–7 d activity frequency. | Used to assist in determining whether the corresponding intensity of physical activity was performed; not used to calculate weekly physical activity volume, average daily physical activity time, or MET-min/week. |
| Moderate-intensity physical activity duration | 4. On one of these days, how much time do you usually spend in moderate-intensity physical activity? (If the duration differs from day to day, please estimate the average number of hours and minutes, or use records from a fitness tracker if available.) | “____ hours per day”; “____ minutes (less than 60 minutes)” | Hours and minutes were converted to minutes; participants who did not engage in the corresponding activity were coded as 0 min. The final variable was interpreted as self-reported moderate-intensity physical activity duration on a typical activity day, min/typical activity day. |
| Light-intensity physical activity participation | 5. During the past 7 days, on how many days did you walk (for at least 10 minutes at a time) or engage in light-intensity activity? (Definition: activities that do not make you feel tired or sweaty, but during which you are active—not sitting.) Please include: transportation: walking from the dormitory to the classroom building/library/cafeteria, walking to the campus gate, walking home from a subway station; leisure: walking around campus, shopping, walking a dog; standing activities: conducting experiments while standing, drawing/designing while standing, cooking/washing dishes while standing; light activities: stretching, organizing a desk, making a bed. | The original database retained only categorical coding for this item and did not retain the complete number of days required to calculate a 0–7 d activity frequency. | Used to assist in determining whether questionnaire-defined light-intensity physical activity was performed. Because the item includes different activities such as walking, standing, and stretching, it was treated as a questionnaire-defined self-reported light-intensity physical activity indicator rather than LPA validated against objective energy-expenditure thresholds. |
| Light-intensity physical activity duration | 6. On one of these days, how much time do you usually spend walking or engaging in light-intensity activity? (If the duration differs from day to day, please estimate the average number of hours and minutes, or use records from a fitness tracker if available.) | “____ hours per day”; “____ minutes (less than 60 minutes)” | Hours and minutes were converted to minutes; participants who did not engage in the corresponding activity were coded as 0 min. The final variable was interpreted as self-reported light-intensity physical activity duration on a typical activity day, min/typical activity day. |
| Sedentary behavior | 7. During the past 7 days, how much time per day did you usually spend “sitting” or “lying down” (excluding sleep)? Study/work-related: attending classes, self-study, doing homework, using a computer to search for information; screen-based entertainment: watching short videos, playing games, watching shows, non-study-related Internet use; other sedentary activities: riding in a vehicle, riding an electric scooter while seated, eating, chatting while seated, or sitting idly. | “____ hours per day”; “____ minutes (less than 60 minutes)” | Converted to min/d using hours × 60 + minutes. Only sedentary time directly reported in the questionnaire was used; unmeasured remaining time was not assigned to sedentary behavior, and sedentary time was not back-calculated from physical activity or sleep duration. |
| Sleep duration | 4. During the past month, how many hours of actual sleep did you get each night? (This is not the same as time spent in bed.) | The original database stored this item as categorical codes; the cleaned dataset contains four sleep-duration categories: ≥7 h, 6 to <7 h, 5 to <6 h, and <5 h. | All four categories were retained for descriptive analyses; for the primary regression analyses, they were collapsed into ≥7 h and <7 h, with ≥7 h as the reference category. No arbitrary midpoint was assigned to the categories, and they were not converted into continuous minutes. |

**Note:** The physical activity items were adapted with reference to the general structure and activity-intensity concepts of the IPAQ-SF and were tailored to daily activity contexts among university students. However, these items were not standard IPAQ-SF items and were not scored using the standard IPAQ-SF scoring protocol. Activity intensity was classified according to the subjective intensity descriptions and activity examples provided in the original questionnaire rather than reclassified on the basis of individually measured energy expenditure or MET values. Therefore, the vigorous-, moderate-, and light-intensity physical activity indicators in this study should not be considered directly equivalent to measures obtained using the standard IPAQ-SF, other validated standardized questionnaires, or accelerometers. The original database did not retain complete activity-frequency information required to calculate weekly physical activity volume; therefore, the three physical activity duration variables represent only participants’ self-reported activity time on a typical activity day, and weekly physical activity volume, average daily physical activity duration, and MET-min/week cannot be calculated. In the regression models, “per 30 min” represents the difference in outcome score associated with a 30-min difference in duration on a typical activity day, rather than an additional 30 min of physical activity per average day or per week. The original light-intensity physical activity item included walking, standing, stretching, and other low-load activities. Because static standing does not necessarily meet an energy-expenditure-based definition of light-intensity physical activity, this indicator may involve activity-intensity construct misclassification and should be interpreted as a questionnaire-defined self-reported light-intensity physical activity indicator. Sedentary behavior represents self-reported usual daily time spent sitting or lying during the previous 7 days, excluding sleep. The sleep item asked about actual nightly sleep duration during the previous month and explicitly stated that this was not equivalent to time in bed.

**Supplementary Table S2. Missingness in Exposure Variables, Outcome Variables, and Covariates**

| **Variable category** | **Variable** | **Valid observations, n** | **Missing/set to missing, n (%)** | **Known reason for missingness** |
| --- | --- | --- | --- | --- |
| Exposure variables | Vigorous-intensity physical activity | 775 | 1(0.1) | One activity-duration entry was ambiguous and could not be reliably converted to minutes; it was therefore set to missing. |
|  | Moderate-intensity physical activity | 776 | 0(0.0) | No missing values |
|  | Light-intensity physical activity | 773 | 3(0.4) | Three duration reports exceeded 1,440 min/typical activity day and were set to missing according to the prespecified data-cleaning rules. |
|  | Sedentary behavior | 772 | 4(0.5) | One time field could not be reliably parsed, and three reported total sedentary times exceeded 1,440 min/d; all were set to missing. |
|  | Sleep duration | 776 | 0(0.0) | No missing values |
| Outcome variables | Depression score | 776 | 0(0.0) | No missing values |
|  | Anxiety score | 776 | 0(0.0) | No missing values |
|  | Stress score | 776 | 0(0.0) | No missing values |
|  | Problematic Internet use score | 776 | 0(0.0) | No missing values |
| Covariates | Age | 776 | 0(0.0) | No missing values |
|  | Body mass index (BMI) | 773 | 3(0.4) | Two height records and one weight record were outside the prespecified valid ranges, preventing BMI calculation. |
|  | Admission year | 767 | 9(1.2) | Admission year could not be reliably identified from major and class information. |

**Note: Missing proportions were calculated using the full sample (n=776) as the denominator. For participants who did not engage in the corresponding intensity of physical activity, duration was coded as 0 min and was not considered missing. Time records that could not be reliably parsed, negative values, and single reported time values >1,440 min were treated as missing. All statistical models used complete-case analysis, with no imputation of missing values. Exclusion of female participants from the primary inferential analyses because those analyses were restricted to males represents an analytical sample restriction rather than missing data. As specified in the Methods, time values that could not be reliably converted and values >1,440 min were treated as missing, and complete-case analysis was used.**

**Supplementary Table S3. Upper-Tail Distribution of Physical Activity Duration Before Truncation**

| **Physical activity indicator** | **Valid sample size, n** | **>180 min/typical activity day, n (%)** | **P95, min** | **P99, min** | **Maximum, min** |
| --- | --- | --- | --- | --- | --- |
| Vigorous-intensity physical activity | 775 | 27(3.5) | 172.7 | 240 | 360 |
| Moderate-intensity physical activity | 776 | 30(3.9) | 180 | 247.5 | 360 |
| Light-intensity physical activity | 773 | 127(16.4) | 301.4 | 482.8 | 960 |

**Note:** Physical activity duration represents participants’ self-reported activity time on a typical activity day. P95 and P99 denote the 95th and 99th percentiles, respectively. In the primary analysis, values >180 min/typical activity day were truncated to 180 min. A total of 150 participants (19.3%) had at least one physical activity indicator >180 min/typical activity day. Because the same participant could have more than one physical activity indicator exceeding 180 min, the counts across indicators should not be summed directly.

**Supplementary Table S4. Fully Adjusted GEE Analysis of ln(x+1)-Transformed DASS-21 Scores**

| **Outcome** | **Behavioral indicator** | **B(95%CI)** | **P value** | **q value** |
| --- | --- | --- | --- | --- |
| Depression | Vigorous-intensity physical activity, per 30 min/typical activity day | −0.042(−0.087–0.002) | 0.059 | 0.127 |
|  | Moderate-intensity physical activity, per 30 min/typical activity day | 0.019(−0.027–0.065) | 0.421 | 0.526 |
|  | Light-intensity physical activity, per 30 min/typical activity day | 0.005(−0.027–0.037) | 0.759 | 0.768 |
|  | Sedentary behavior, per 60 min/d | 0.004(−0.016–0.025) | 0.678 | 0.768 |
|  | Sleep duration <7 h (reference: ≥7 h) | 0.189(0.062–0.316) | 0.004 | 0.013 |
| Anxiety | Vigorous-intensity physical activity, per 30 min/typical activity day | −0.084(−0.135–−0.033) | 0.001 | 0.006 |
|  | Moderate-intensity physical activity, per 30 min/typical activity day | 0.025(−0.027–0.077) | 0.354 | 0.493 |
|  | Light-intensity physical activity, per 30 min/typical activity day | −0.006(−0.043–0.032) | 0.768 | 0.768 |
|  | Sedentary behavior, per 60 min/d | 0.021(−0.000–0.041) | 0.054 | 0.127 |
|  | Sleep duration <7 h (reference: ≥7 h) | 0.212(0.091–0.332) | <0.001 | 0.004 |
| Stress | Vigorous-intensity physical activity, per 30 min/typical activity day | −0.057(−0.107–−0.006) | 0.027 | 0.082 |
|  | Moderate-intensity physical activity, per 30 min/typical activity day | 0.027(−0.024–0.077) | 0.301 | 0.493 |
|  | Light-intensity physical activity, per 30 min/typical activity day | −0.013(−0.041–0.015) | 0.361 | 0.493 |
|  | Sedentary behavior, per 60 min/d | 0.020(−0.005–0.045) | 0.117 | 0.219 |
|  | Sleep duration <7 h (reference: ≥7 h) | 0.227(0.098–0.355) | <0.001 | 0.004 |

**Note:** The primary inferential analysis was restricted to male participants. Depression, anxiety, and stress scores were each transformed using ln(x+1). The model included 756 male participants and 48 class clusters, adjusted for age, body mass index, and admission year, and simultaneously included vigorous-, moderate-, and light-intensity physical activity, sedentary behavior, and sleep duration. B denotes the unstandardized regression coefficient and CI denotes confidence interval. q values were obtained by applying the Benjamini–Hochberg procedure for FDR correction across the 15 behavior–outcome associations.

**Supplementary Table S5. Fully Adjusted GEE Analysis Using DASS-21 Symptom Screening Thresholds**

| **Outcome** | **Behavioral indicator** | **PR(95%CI)** | **P value** | **q value** |
| --- | --- | --- | --- | --- |
| Above depression screening threshold | Vigorous-intensity physical activity, per 30 min/typical activity day | 0.831(0.699–0.987) | 0.035 | 0.173 |
|  | Moderate-intensity physical activity, per 30 min/typical activity day | 1.079(0.871–1.336) | 0.486 | 0.663 |
|  | Light-intensity physical activity, per 30 min/typical activity day | 0.981(0.820–1.172) | 0.829 | 0.957 |
|  | Sedentary behavior, per 60 min/d | 0.995(0.909–1.089) | 0.920 | 0.986 |
|  | Sleep duration <7 h (reference: ≥7 h) | 2.286(1.264–4.135) | 0.006 | 0.047 |
| Above anxiety screening threshold | Vigorous-intensity physical activity, per 30 min/typical activity day | 0.810(0.719–0.912) | <0.001 | 0.008 |
|  | Moderate-intensity physical activity, per 30 min/typical activity day | 1.154(1.002–1.328) | 0.046 | 0.173 |
|  | Light-intensity physical activity, per 30 min/typical activity day | 0.977(0.885–1.080) | 0.654 | 0.818 |
|  | Sedentary behavior, per 60 min/d | 1.037(0.997–1.079) | 0.069 | 0.208 |
|  | Sleep duration <7 h (reference: ≥7 h) | 1.434(0.886–2.322) | 0.142 | 0.356 |
| Above stress screening threshold | Vigorous-intensity physical activity, per 30 min/typical activity day | 0.829(0.600–1.145) | 0.255 | 0.426 |
|  | Moderate-intensity physical activity, per 30 min/typical activity day | 1.310(0.834–2.056) | 0.241 | 0.426 |
|  | Light-intensity physical activity, per 30 min/typical activity day | 1.000(0.819–1.222) | 0.998 | 0.998 |
|  | Sedentary behavior, per 60 min/d | 1.061(0.940–1.198) | 0.336 | 0.504 |
|  | Sleep duration <7 h (reference: ≥7 h) | 2.523(0.661–9.628) | 0.175 | 0.376 |

**Note:** The primary inferential analysis was restricted to male participants. Scores above the depression, anxiety, and stress screening thresholds were defined as DASS-21 subscale scores ≥10, ≥8, and ≥15, respectively. In the fully adjusted analysis of the male primary inferential sample, 51, 86, and 13 participants were above the depression, anxiety, and stress screening thresholds, respectively. GEE models with a Poisson distribution, log link, independent working correlation structure, and robust sandwich standard errors were used to estimate prevalence ratios (PRs) and 95% CIs. Models adjusted for age, body mass index, and admission year and simultaneously included vigorous-, moderate-, and light-intensity physical activity, sedentary behavior, and sleep duration. Vigorous-, moderate-, and light-intensity physical activity were scaled per 30 min/typical activity day, sedentary behavior per 60 min/d, and sleep duration used ≥7 h as the reference category. q values were obtained by applying the Benjamini–Hochberg procedure for FDR correction across the 15 prespecified behavior–outcome associations. Because the number of participants above some screening thresholds was small, particularly only 13 for stress, the corresponding estimates have limited precision. These results should therefore be interpreted cautiously and are presented only as sensitivity analyses for alternative outcome definitions and modeling approaches, rather than as confirmation of the robustness of the primary findings.

**Supplementary Table S6. Fully Adjusted GEE Analysis Using Untruncated Physical Activity Duration**

| **Outcome** | **Behavioral indicator** | **B(95%CI)** | **P value** | **q value** |
| --- | --- | --- | --- | --- |
| Depression | Vigorous-intensity physical activity, per 30 min/typical activity day | −0.210(−0.402–−0.018) | 0.032 | 0.079 |
|  | Moderate-intensity physical activity, per 30 min/typical activity day | 0.129(−0.083–0.340) | 0.234 | 0.335 |
|  | Light-intensity physical activity, per 30 min/typical activity day | 0.015(−0.105–0.136) | 0.803 | 0.892 |
|  | Sedentary behavior, per 60 min/d | −0.008(−0.088–0.072) | 0.848 | 0.893 |
|  | Sleep duration <7 h (reference: ≥7 h) | 0.908(0.341–1.475) | 0.002 | 0.007 |
| Anxiety | Vigorous-intensity physical activity, per 30 min/typical activity day | −0.343(−0.538–−0.149) | <0.001 | 0.004 |
|  | Moderate-intensity physical activity, per 30 min/typical activity day | 0.233(−0.027–0.493) | 0.079 | 0.158 |
|  | Light-intensity physical activity, per 30 min/typical activity day | −0.031(−0.132–0.069) | 0.543 | 0.638 |
|  | Sedentary behavior, per 60 min/d | 0.056(−0.024–0.136) | 0.172 | 0.265 |
|  | Sleep duration <7 h (reference: ≥7 h) | 0.982(0.443–1.521) | <0.001 | 0.004 |
| Stress | Vigorous-intensity physical activity, per 30 min/typical activity day | −0.278(−0.513–−0.043) | 0.020 | 0.058 |
|  | Moderate-intensity physical activity, per 30 min/typical activity day | 0.172(−0.053–0.397) | 0.134 | 0.224 |
|  | Light-intensity physical activity, per 30 min/typical activity day | −0.002(−0.151–0.148) | 0.984 | 0.984 |
|  | Sedentary behavior, per 60 min/d | 0.085(−0.017–0.186) | 0.103 | 0.188 |
|  | Sleep duration <7 h (reference: ≥7 h) | 1.092(0.518–1.667) | <0.001 | 0.004 |
| Problematic Internet use | Vigorous-intensity physical activity, per 30 min/typical activity day | −0.381(−0.786–0.025) | 0.066 | 0.146 |
|  | Moderate-intensity physical activity, per 30 min/typical activity day | −0.167(−0.571–0.237) | 0.417 | 0.557 |
|  | Light-intensity physical activity, per 30 min/typical activity day | −0.065(−0.273–0.143) | 0.539 | 0.638 |
|  | Sedentary behavior, per 60 min/d | 0.332(0.115–0.549) | 0.003 | 0.009 |
|  | Sleep duration <7 h (reference: ≥7 h) | 2.209(0.830–3.588) | 0.002 | 0.007 |

**Note:** The primary inferential analysis was restricted to male participants. The model included 756 male participants and 48 class clusters, adjusted for age, body mass index, and admission year, and simultaneously included vigorous-, moderate-, and light-intensity physical activity, sedentary behavior, and sleep duration. The original, untruncated duration values were used for all three physical activity indicators. B denotes the unstandardized regression coefficient and CI denotes confidence interval. q values were obtained by applying the Benjamini–Hochberg procedure for FDR correction across the 20 behavior–outcome associations.

**Supplementary Table S7. Fully Adjusted GEE Analysis After Excluding Participants With Any Physical Activity Duration >180 min/Typical Activity Day**

| **Outcome** | **Behavioral indicator** | **B(95%CI)** | **P value** | **q value** |
| --- | --- | --- | --- | --- |
| Depression | Vigorous-intensity physical activity, per 30 min/typical activity day | −0.159(−0.377–0.060) | 0.154 | 0.280 |
|  | Moderate-intensity physical activity, per 30 min/typical activity day | 0.018(−0.221–0.257) | 0.882 | 0.883 |
|  | Light-intensity physical activity, per 30 min/typical activity day | 0.044(−0.125–0.212) | 0.611 | 0.873 |
|  | Sedentary behavior, per 60 min/d | 0.019(−0.076–0.114) | 0.701 | 0.876 |
|  | Sleep duration <7 h (reference: ≥7 h) | 0.838(0.152–1.524) | 0.017 | 0.048 |
| Anxiety | Vigorous-intensity physical activity, per 30 min/typical activity day | −0.295(−0.513–−0.077) | 0.008 | 0.032 |
|  | Moderate-intensity physical activity, per 30 min/typical activity day | 0.078(−0.155–0.311) | 0.514 | 0.796 |
|  | Light-intensity physical activity, per 30 min/typical activity day | 0.047(−0.096–0.191) | 0.518 | 0.796 |
|  | Sedentary behavior, per 60 min/d | 0.098(0.006–0.189) | 0.036 | 0.090 |
|  | Sleep duration <7 h (reference: ≥7 h) | 1.065(0.379–1.751) | 0.002 | 0.016 |
| Stress | Vigorous-intensity physical activity, per 30 min/typical activity day | −0.197(−0.400–0.006) | 0.057 | 0.126 |
|  | Moderate-intensity physical activity, per 30 min/typical activity day | −0.024(−0.343–0.295) | 0.883 | 0.883 |
|  | Light-intensity physical activity, per 30 min/typical activity day | 0.037(−0.129–0.204) | 0.661 | 0.876 |
|  | Sedentary behavior, per 60 min/d | 0.148(0.027–0.268) | 0.016 | 0.048 |
|  | Sleep duration <7 h (reference: ≥7 h) | 1.142(0.451–1.833) | 0.001 | 0.012 |
| Problematic Internet use | Vigorous-intensity physical activity, per 30 min/typical activity day | −0.081(−0.627–0.465) | 0.771 | 0.883 |
|  | Moderate-intensity physical activity, per 30 min/typical activity day | −0.542(−1.155–0.070) | 0.083 | 0.165 |
|  | Light-intensity physical activity, per 30 min/typical activity day | −0.055(−0.479–0.368) | 0.798 | 0.883 |
|  | Sedentary behavior, per 60 min/d | 0.458(0.212–0.703) | <0.001 | 0.005 |
|  | Sleep duration <7 h (reference: ≥7 h) | 2.206(0.715–3.698) | 0.004 | 0.019 |

**Note:** The primary inferential analysis was restricted to male participants. Among the 774 male participants, 150 (19.4%) had at least one physical activity indicator >180 min/typical activity day. After excluding participants whose typical-day duration of vigorous-, moderate-, or light-intensity physical activity exceeded 180 min, the fully adjusted model included 608 male participants and 48 class clusters. The model adjusted for age, body mass index, and admission year and simultaneously included the five behavioral indicators. B denotes the unstandardized regression coefficient and CI denotes confidence interval. q values were obtained by applying the Benjamini–Hochberg procedure for FDR correction across the 20 behavior–outcome associations.

**Supplementary Table S8. Exploratory Nonlinear Analysis of Quadratic Terms for Continuous Behavioral Indicators**

| **Behavioral quadratic term** | **Outcome** | **B (quadratic term) (95% CI)** | **P value** | **q value** |
| --- | --- | --- | --- | --- |
| Vigorous-intensity physical activity² | Depression | −0.011(−0.096–0.075) | 0.805 | 0.831 |
|  | Anxiety | −0.044(−0.146–0.058) | 0.399 | 0.699 |
|  | Stress | −0.021(−0.138–0.096) | 0.723 | 0.831 |
|  | Problematic Internet use | −0.147(−0.355–0.062) | 0.168 | 0.538 |
| Moderate-intensity physical activity² | Depression | 0.038(−0.070–0.145) | 0.490 | 0.699 |
|  | Anxiety | −0.012(−0.124–0.099) | 0.831 | 0.831 |
|  | Stress | 0.051(−0.086–0.188) | 0.465 | 0.699 |
|  | Problematic Internet use | 0.035(−0.242–0.313) | 0.802 | 0.831 |
| Light-intensity physical activity² | Depression | −0.083(−0.146–−0.019) | 0.011 | 0.073 |
|  | Anxiety | −0.122(−0.184–−0.061) | <0.001 | 0.001 |
|  | Stress | −0.104(−0.187–−0.021) | 0.014 | 0.073 |
|  | Problematic Internet use | −0.123(−0.368–0.122) | 0.326 | 0.699 |
| Sedentary behavior² | Depression | −0.005(−0.012–0.003) | 0.238 | 0.635 |
|  | Anxiety | −0.004(−0.015–0.008) | 0.524 | 0.699 |
|  | Stress | −0.011(−0.025–0.003) | 0.127 | 0.507 |
|  | Problematic Internet use | −0.013(−0.043–0.017) | 0.408 | 0.699 |

**Note:** The primary inferential analysis was restricted to male participants. Vigorous-, moderate-, and light-intensity physical activity were first rescaled per 30 min/typical activity day, and sedentary behavior was rescaled per 60 min/d; the corresponding squared terms were then constructed. In each model, one quadratic term for a continuous behavioral indicator was added to the primary fully adjusted GEE model. B denotes the regression coefficient for the corresponding quadratic term. A total of 16 quadratic-term tests were conducted, and q values were obtained by applying the Benjamini–Hochberg procedure for FDR correction across all 16 tests. These analyses were exploratory and were not used as the basis for primary statistical inference.

**Supplementary Table S9. Fully Adjusted GEE Sensitivity Analysis Restricted to Participants Who Answered Both Instructional Attention-Check Items Correctly**

| **Outcome** | **Behavioral indicator** | **B(95%CI)** | **P value** | **q value** |
| --- | --- | --- | --- | --- |
| Depression | Vigorous-intensity physical activity, per 30 min/typical activity day | −0.194(−0.425–0.037) | 0.101 | 0.241 |
|  | Moderate-intensity physical activity, per 30 min/typical activity day | 0.092(−0.140–0.324) | 0.437 | 0.583 |
|  | Light-intensity physical activity, per 30 min/typical activity day | 0.007(−0.110–0.125) | 0.904 | 0.911 |
|  | Sedentary behavior, per 60 min/d | −0.005(−0.089–0.080) | 0.911 | 0.911 |
|  | Sleep duration <7 h (reference: ≥7 h) | 0.846(0.257–1.436) | 0.005 | 0.016 |
| Anxiety | Vigorous-intensity physical activity, per 30 min/typical activity day | −0.329(−0.554–−0.104) | 0.004 | 0.016 |
|  | Moderate-intensity physical activity, per 30 min/typical activity day | 0.129(−0.111–0.369) | 0.294 | 0.419 |
|  | Light-intensity physical activity, per 30 min/typical activity day | −0.017(−0.137–0.102) | 0.777 | 0.863 |
|  | Sedentary behavior, per 60 min/d | 0.058(−0.031–0.146) | 0.204 | 0.334 |
|  | Sleep duration <7 h (reference: ≥7 h) | 0.913(0.372–1.455) | <0.001 | 0.007 |
| Stress | Vigorous-intensity physical activity, per 30 min/typical activity day | −0.254(−0.535–0.028) | 0.077 | 0.221 |
|  | Moderate-intensity physical activity, per 30 min/typical activity day | 0.082(−0.242–0.407) | 0.619 | 0.774 |
|  | Light-intensity physical activity, per 30 min/typical activity day | −0.023(−0.174–0.128) | 0.765 | 0.863 |
|  | Sedentary behavior, per 60 min/d | 0.083(−0.026–0.191) | 0.134 | 0.244 |
|  | Sleep duration <7 h (reference: ≥7 h) | 1.038(0.419–1.656) | 0.001 | 0.007 |
| Problematic Internet use | Vigorous-intensity physical activity, per 30 min/typical activity day | −0.297(−0.769–0.174) | 0.217 | 0.334 |
|  | Moderate-intensity physical activity, per 30 min/typical activity day | −0.341(−0.757–0.075) | 0.108 | 0.241 |
|  | Light-intensity physical activity, per 30 min/typical activity day | −0.262(−0.595–0.071) | 0.123 | 0.244 |
|  | Sedentary behavior, per 60 min/d | 0.314(0.117–0.512) | 0.002 | 0.009 |
|  | Sleep duration <7 h (reference: ≥7 h) | 2.590(1.108–4.073) | <0.001 | 0.007 |

**Note:** This sensitivity analysis was restricted to male participants who answered both instructional attention-check items correctly (n=688); the fully adjusted model ultimately included 674 participants and 46 class clusters. The model adjusted for age, body mass index, and admission year and simultaneously included vigorous-, moderate-, and light-intensity physical activity, sedentary behavior, and sleep duration. B denotes the unstandardized regression coefficient and CI denotes confidence interval. q values were obtained by applying the Benjamini–Hochberg procedure for FDR correction across the 20 behavior–outcome associations.

**Supplementary Table S10. Fully Adjusted GEE Sensitivity Analysis Using an Independent Working Correlation Structure**

| **Outcome** | **Behavioral indicator** | **B(95%CI)** | **P value** | **q value** |
| --- | --- | --- | --- | --- |
| Depression | Vigorous-intensity physical activity, per 30 min/typical activity day | −0.225(−0.434–−0.016) | 0.035 | 0.088 |
|  | Moderate-intensity physical activity, per 30 min/typical activity day | 0.128(−0.092–0.348) | 0.254 | 0.339 |
|  | Light-intensity physical activity, per 30 min/typical activity day | 0.009(−0.127–0.145) | 0.896 | 0.896 |
|  | Sedentary behavior, per 60 min/d | −0.006(−0.083–0.071) | 0.874 | 0.896 |
|  | Sleep duration <7 h (reference: ≥7 h) | 0.914(0.347–1.481) | 0.002 | 0.006 |
| Anxiety | Vigorous-intensity physical activity, per 30 min/typical activity day | −0.356(−0.562–−0.150) | <0.001 | 0.005 |
|  | Moderate-intensity physical activity, per 30 min/typical activity day | 0.181(−0.049–0.410) | 0.122 | 0.235 |
|  | Light-intensity physical activity, per 30 min/typical activity day | −0.018(−0.153–0.116) | 0.789 | 0.876 |
|  | Sedentary behavior, per 60 min/d | 0.054(−0.027–0.134) | 0.191 | 0.313 |
|  | Sleep duration <7 h (reference: ≥7 h) | 0.981(0.440–1.523) | <0.001 | 0.004 |
| Stress | Vigorous-intensity physical activity, per 30 min/typical activity day | −0.280(−0.536–−0.023) | 0.032 | 0.088 |
|  | Moderate-intensity physical activity, per 30 min/typical activity day | 0.147(−0.146–0.441) | 0.326 | 0.407 |
|  | Light-intensity physical activity, per 30 min/typical activity day | −0.021(−0.157–0.114) | 0.759 | 0.876 |
|  | Sedentary behavior, per 60 min/d | 0.085(−0.016–0.186) | 0.099 | 0.219 |
|  | Sleep duration <7 h (reference: ≥7 h) | 1.109(0.529–1.690) | <0.001 | 0.004 |
| Problematic Internet use | Vigorous-intensity physical activity, per 30 min/typical activity day | −0.338(−0.773–0.098) | 0.129 | 0.235 |
|  | Moderate-intensity physical activity, per 30 min/typical activity day | −0.285(−0.748–0.178) | 0.227 | 0.324 |
|  | Light-intensity physical activity, per 30 min/typical activity day | −0.199(−0.507–0.108) | 0.204 | 0.313 |
|  | Sedentary behavior, per 60 min/d | 0.327(0.112–0.543) | 0.003 | 0.010 |
|  | Sleep duration <7 h (reference: ≥7 h) | 2.267(0.867–3.668) | 0.002 | 0.006 |

**Note:** The primary inferential analysis was restricted to male participants. In this sensitivity analysis, the fully adjusted GEE model was refitted using an independent working correlation structure and included 756 male participants and 48 class clusters. The model adjusted for age, body mass index, and admission year and simultaneously included the five behavioral indicators. B denotes the unstandardized regression coefficient and CI denotes confidence interval. q values were obtained by applying the Benjamini–Hochberg procedure for FDR correction across the 20 behavior–outcome associations.

**Supplementary Table S11. Fully Adjusted GEE Analysis After Excluding Participants Aged <18 Years**

| **Outcome** | **Behavioral indicator** | **B(95%CI)** | **P value** | **q value** |
| --- | --- | --- | --- | --- |
| Depression | Vigorous-intensity physical activity, per 30 min/typical activity day | −0.235(−0.445–−0.025) | 0.028 | 0.071 |
|  | Moderate-intensity physical activity, per 30 min/typical activity day | 0.128(−0.092–0.349) | 0.255 | 0.34 |
|  | Light-intensity physical activity, per 30 min/typical activity day | 0.011(−0.126–0.148) | 0.872 | 0.917 |
|  | Sedentary behavior, per 60 min/d | −0.004(−0.082–0.074) | 0.919 | 0.919 |
|  | Sleep duration <7 h (reference: ≥7 h) | 0.915(0.343–1.486) | 0.002 | **0.007** |
| Anxiety | Vigorous-intensity physical activity, per 30 min/typical activity day | −0.367(−0.572–−0.162) | <0.001 | **0.003** |
|  | Moderate-intensity physical activity, per 30 min/typical activity day | 0.183(−0.045–0.410) | 0.115 | 0.21 |
|  | Light-intensity physical activity, per 30 min/typical activity day | −0.015(−0.150–0.120) | 0.826 | 0.917 |
|  | Sedentary behavior, per 60 min/d | 0.054(−0.027–0.135) | 0.191 | 0.295 |
|  | Sleep duration <7 h (reference: ≥7 h) | 0.995(0.450–1.541) | <0.001 | **0.003** |
| Stress | Vigorous-intensity physical activity, per 30 min/typical activity day | −0.299(−0.556–−0.043) | 0.022 | 0.064 |
|  | Moderate-intensity physical activity, per 30 min/typical activity day | 0.151(−0.142–0.444) | 0.312 | 0.39 |
|  | Light-intensity physical activity, per 30 min/typical activity day | −0.029(−0.163–0.105) | 0.672 | 0.79 |
|  | Sedentary behavior, per 60 min/d | 0.087(−0.013–0.186) | 0.088 | 0.183 |
|  | Sleep duration <7 h (reference: ≥7 h) | 1.115(0.537–1.692) | <0.001 | **0.003** |
| Problematic Internet use | Vigorous-intensity physical activity, per 30 min/typical activity day | −0.379(−0.819–0.061) | 0.092 | 0.183 |
|  | Moderate-intensity physical activity, per 30 min/typical activity day | −0.272(−0.734–0.189) | 0.247 | 0.34 |
|  | Light-intensity physical activity, per 30 min/typical activity day | −0.206(−0.505–0.094) | 0.179 | 0.295 |
|  | Sedentary behavior, per 60 min/d | 0.329(0.113–0.545) | 0.003 | **0.01** |
|  | Sleep duration <7 h (reference: ≥7 h) | 2.246(0.833–3.658) | 0.002 | **0.007** |

**Note:** The primary inferential analysis was restricted to male participants. In this sensitivity analysis, four participants aged <18 years were additionally excluded from the male primary inferential sample, leaving 752 participants and 48 class clusters in the fully adjusted model. The model used Gaussian GEE with an exchangeable working correlation structure and robust sandwich standard errors, adjusted for age, body mass index, and admission year, and simultaneously included vigorous-, moderate-, and light-intensity physical activity, sedentary behavior, and sleep duration. All three physical activity indicators represent self-reported activity duration on a typical activity day; under the primary-analysis rule, values >180 min/typical activity day were truncated to 180 min. Physical activity coefficients were scaled per 30 min/typical activity day, sedentary behavior per 60 min/d, and sleep duration used ≥7 h as the reference category. B denotes the unstandardized regression coefficient and CI denotes confidence interval. q values are the Benjamini–Hochberg FDR-corrected results for the 20 behavior–outcome associations. All estimates represent cross-sectional statistical associations and are not interpreted causally.

**Supplementary Figure 1. Distribution of Depression Scores**

**Supplementary Figure 2. Distribution of Anxiety Scores**

**Supplementary Figure 3. Distribution of Stress Scores**

**Supplementary Figure 4. Distribution of Problematic Internet Use Scores**

**Supplementary Figure 5. Pearson Residuals Versus Fitted Values for the GEE Depression Model**

**Supplementary Figure 6. Pearson Residuals Versus Fitted Values for the GEE Anxiety Model**

**Supplementary Figure 7. Pearson Residuals Versus Fitted Values for the GEE Stress Model**

**Supplementary Figure 8. Pearson Residuals Versus Fitted Values for the GEE Problematic Internet Use Model**
